# Chemical applicability and computation of K-Banhatti indices for benzenoid hydrocarbons and triazine-based covalent organic frameworks

**DOI:** 10.1038/s41598-023-45061-y

**Published:** 2023-10-18

**Authors:** M. C. Shanmukha, Rashad Ismail, K. J. Gowtham, A. Usha, Muhammad Azeem, Esmail Hassan Abdullatif Al-Sabri

**Affiliations:** 1 Department of Mathematics, PES Institute of Technology and Management, Shivamogga, 577204 India; 2 Department of Mathematics, Faculty of Science and Arts, Mahayl Assir, King Khalid University, Abha, Saudi Arabia; 3 Department of Mathematics and Computer, Faculty of Science, Ibb University, Ibb, 70270 Yemen; 4 Department of Mathematics, University College of Science, Tumkur University, Tumakuru, 572103 India; 5 Department of Mathematics, Alliance School of Applied Mathematics, Alliance University, Bangalore, 562106 India; 6 Department of Mathematics, Riphah International University Lahore, Lahore, Pakistan

**Keywords:** Biochemistry, Drug discovery, Chemistry, Engineering, Materials science, Mathematics and computing, Physics

## Abstract

The novel applications in chemistry include the mathematical models of molecular structure of the compounds which has numerous findings in this area that refers to mathematical chemistry. Topological descriptors play a major role in QSAR/QSPR studies that analyses the biological and physicochemical properties of the compounds. In the recent times, a new type of topological descriptors are proposed, called K-Banhatti indices. In this study the chemical applicability of K-Banhatti indices are examined for benzenoid hydrocarbons (derivatives of benzene). These indices have shown remarkable results through the study of statistical analysis. Subsequently, triazine-based covalent organic frameworks (CoF’s) are studied for which $B_1(G)$, $B_2(G)$, $HB_1(G)$, $HB_2(G)$, ${}^mB_1(G)$, ${}^mB_2(G)$, and *HB*(*G*) of a graph G are computed.

## Introduction

The structure of the molecule can be quantified by the usage of topological descriptors. There are varied applications in chemistry and these quantities are derived from their molecular structure. The chemical information obtained from the molecular descriptors vary for different algorithms proposed according to its respective definitions. Using the algorithm of a particular molecular descriptor, a detailed description of a molecule can be obtained. The selection of a molecular descriptor depends on the problem on which it is applied. The key technique is in encoding the information obtained from molecular descriptors using the structure of the molecule^1^.

The modelling and forecasting of physicochemical and biological properties of molecules is aimed in the studies of Quantitative structure–property relationships (QSPR). Statistical and mathematical tools are used to extract every possible information about a compound using the help of chemometrics. The variation of physicochemical property of a molecule with respect to the topological index can be described using chemometrics through QSPR. This can replace expensive biological tests conducted in a laboratory, especially when the experiments involve hazardous and toxic materials or unstable compounds. An optimum relationship in predicting the properties of compounds is the basic strategy of QSPR. The performance of this study depends on the description of the molecular structure and their parameters^2^.

To encode the information of a chemical structure, several topological indices were developed. These indices draw attention as they play a significant role in the contributions of QSPR studies^3–6^. The molecular descriptors are used to extract most of the information of a compound using simple and quick computations.

Topological descriptors have a crucial role in QSAR/QSPR studies that analyses the biological and significant properties of the compounds. The mathematical chemistry is a combination of modelling of chemical compound to derive pivotal data through the various tools viz., topological indices, QSAR/QSPR studies and various polynomials^7–12^.

**Figure 1:**
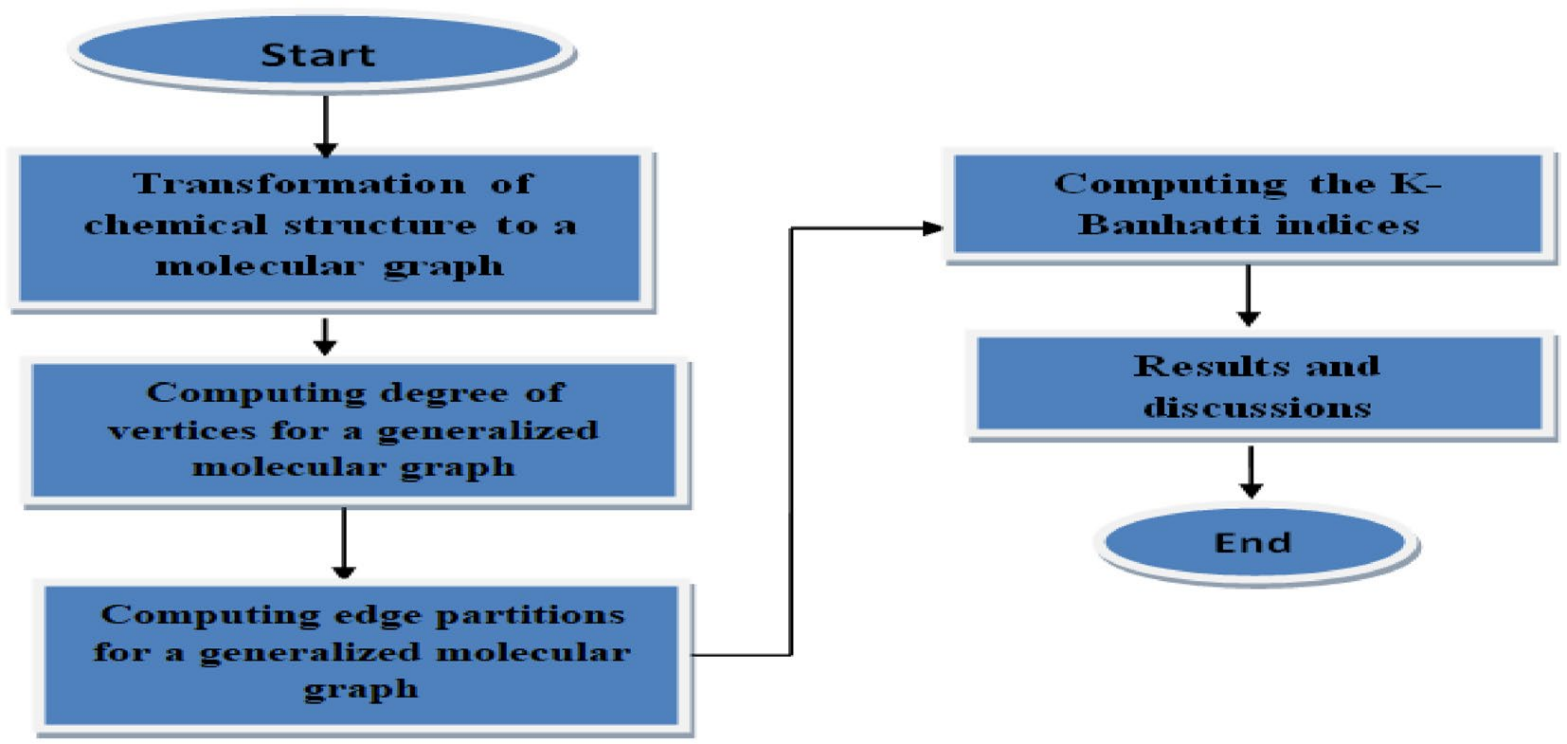
Flowchart to calculate the K-Banhatti indices.

Recently in Refs.^13,14^, Kulli proposed various novel degree-based TIs such as the first K-Banhatti index $(B_1(G))$, the second K-Banhatti index $(B_2(G))$, first K hyper-Banhatti index $(HB_1(G))$, the second K hyper-Banhatti index $(HB_2(G))$, the modified first K-Banhatti index $({}^mB_1(G))$, the modified second K-Banhatti index $({}^mB_2(G))$ and the harmonic K-Banhatti index (*HB*(*G*)) of a graph *G* are defined as

$$\begin{aligned}{} & {} B_1(G)=\sum _{ue\in G}\left( d_u+d_e\right) ,\ \ \ B_2(G)=\sum _{ue\in G}\left( d_u\cdot d_e\right) ,\ \ \ HB_1(G)=\sum _{ue\in G} \left[ d_u+d_e \right] ^2, \\{} & {} HB_2(G)=\sum _{ue\in G}\left[ d_u\cdot d_e \right] ^2,\ {}^mB_1(G)=\sum _{ue\in G}\left( \dfrac{1}{d_u+d_e}\right) ,\ {}^mB_2(G)=\sum _{ue\in G}\left( \dfrac{1}{d_u\cdot d_e}\right) , \end{aligned}$$

and $HB(G)=\sum _{ue\in G}\left( \dfrac{2}{d_u+d_e}\right)$ respectively. And ${ue\in G}$ or $u \sim e$ represents the vertex *u*, and an edge *e* are incident in the graph *G* and numerous studies have been carried out on these indices so far^15–19^. But it is noticed that the chemical applicability of these indices have not been studied yet. Furthermore, the flowchart of the metodology defined above is shown in the Fig. 1. This study pinpoints in examining the chemical applicability of the K-Banhatti indices for benzenoid hydrocarbons and it is noticed that the results are truly remarkable through statistical analysis compared to existing work^20–23^ . Subsequently, triazine-based covalent organic frameworks (TriCF) are studied for which the first K-Banhatti index, the second K-Banhatti index, first K-hyper Banhatti index, the second K-hyper Banhatti index, the modified first K-Banhatti index, the modified second K-Banhatti index, and the harmonic K-Banhatti index of a graph G are computed.

In this paper, consider the simple and connected graph $G=(V,E)$, and the vertex set $V=V(G)$, an edge set $E=E(G)$. The cardinality of the set *V* and *E* are known as the order, and the size of the graph *G* respectively. The cardinality of edges incident to a vertex $u\in V$ is called the vertex degree, represented by $d_u$, $d_e$ is the degree of an edge *e* denoted by, $d_e=d_u+d_v-2$ where, $e=uv$. We use $u \sim e$ for the vertex *u* and an edge *e* are adjacent in the graph *G*^24–26^.

## Chemical applicability of K-Banhatti indices

**Figure 2:**
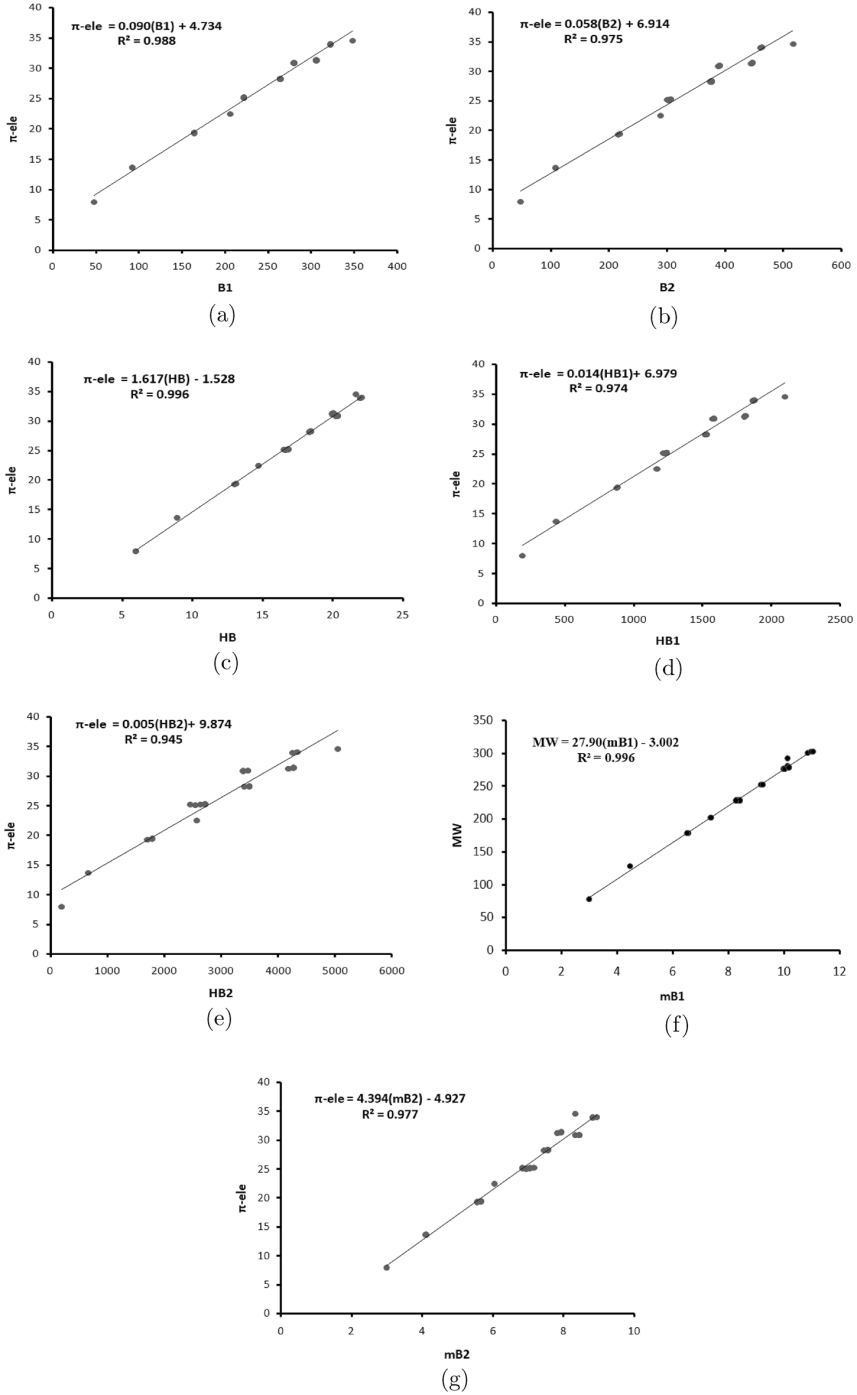
Correlation between (**a**) $\pi -ele$ with $B_1$, (**b**) $\pi -ele$ with $B_2$, (**c**) $\pi -ele$ with *HB*, (**d**) $\pi -ele$ with $HB_1$, (**e**) $\pi -ele$ with $HB_2$, (**f**) *MW* with $^mB_1$, (**g**) $\pi -ele$ with $^mB_2$.

This section concentrates on framing the linear regression model for the properties such as boiling point (BP), enthalpy (E), $\pi$- electron energy ($\pi$-ele), and molecular weight (MW) of benzene derivatives^27,28^ for the considered indices using Tables 1 and 2. It is noticed from Tables 3, 4, 5, 6, 7, 8 and 9 that the regression model of statistical parameters show significant values and the coefficient of correlation *R* with the above four properties show high positive correlation (also see Fig. 2). It is evident from Table 10 that, $B_{1}(G)$, $B_2(G)$, $HB_1(G)$, $HB_2(G)$, ${}^mB_1(G)$, *HB*(*G*) are highly correlated with the $\pi$- electron energy, while ${}^mB_2(G)$ highly correlated with molecular weight.

1. The linear regression models for the first K-Banhatti index($B_1$)
  $$\begin{aligned} BP&=1.68 (\pm 0.053) B_1+ 48.68(\pm 13.68), \\ E&=0.941(\pm 0.081) B_1+ 56.91(\pm 20.84), \\ \pi -\text {ele}&=0.902(\pm 0.0021) B_1+4.734(\pm 0.5516), \\ MW&=0.772( \pm 0.0289)+52.56(\pm 7.4151). \end{aligned}$$
2. The linear regression models for the second K-Banhatti index($B_2$)
  $$\begin{aligned} BP&=1.083(\pm 0.0457)B_2+89.655(\pm 16.5832), \\ E&=0.5992(\pm 0.0589) B_2+82.182(\pm 21.3396), \\ \pi -ele&=0.058(\pm 0.002)B_2+6.913(\pm 0.7475), \\ MW&=0.4956(\pm 0.0244)B_2+71.747(\pm 8.8591). \end{aligned}$$
3. The linear regression models for the first K-hyper Banhatti index($HB_1$)
  $$\begin{aligned} BP&=0.265(\pm 0.0113)HB_1+90.885(\pm 16.6388),\\ E&=0.1469(\pm 0.145)HB_1+82.926(\pm 21.3389),\\ \pi -ele&=0.0142(\pm 0.0005)HB_1+6.979(\pm 0.7518),\\ MW&=0.1215(\pm 0.006)HB_1+72.321(\pm 8.8873). \end{aligned}$$
4. The linear regression models for the second K-hyper Banhatti index($HB_2$)
  $$\begin{aligned} BP&=0.1027(\pm 0.0062)HB_2+145.442(\pm 20.356),\\ E&=0.0559(\pm 0.0065)HB_2+115.865(\pm 21.6844),\\ \pi -ele&=0.0055(\pm 0.0002)HB_2+9.874(\pm 0.9758),\\ MW&=0.0468(\pm 0.0031)HB_2+97.705(\pm 10.4979). \end{aligned}$$
5. The linear regression models for the modified first K-Banhatti index(${}^mB_1$)
  $$\begin{aligned} BP&=60.419(\pm 1.4046){}^mB_1-68.559(\pm 12.674),\\ E&=34.893(\pm 2.0365){}^mB_1-18.295(\pm 18.2957),\\ \pi -ele&=3.2349(\pm 0.0421){}^mB_1-1.529(\pm 0.3802),\\ MW&=27.908(\pm 0.3818){}^mB_1-3.002(\pm 3.4452). \end{aligned}$$
6. The linear regression models for the modified second K-Banhatti index(${}^mB_2$)
  $$\begin{aligned} BP&=82.0515(\pm 3.2353){}^mB_2-131.83(\pm 23.9213),\\ E&=48.3104(\pm 2.3741){}^mB_2-61.5211(\pm 17.553),\\ \pi -ele&=4.3946(\pm 0.1485){}^mB_2-4.927(\pm 1.098),\\ MW&=38.0851(\pm 1.007){}^mB_2-33.5625(\pm 7.4502). \end{aligned}$$
7. The linear regression models for the harmonic K-Banhatti index(*HB*)
  $$\begin{aligned} BP&=30.2102(\pm 0.703)HB-68.5467(\pm 12.6876),\\ E&=17.4474(\pm 1.0182)HB-18.2934(\pm 18.3746),\\ \pi -ele&=1.6175(\pm 0.021)HB-1.5288(\pm 0.3804),\\ MW&=13.9548(\pm 0.1904)HB-3.0001(\pm 3.4359). \end{aligned}$$

**Table 1:** Experimental values (boiling point (BP), enthalpy (E), $\pi$-electron energy ($\pi -{\text {ele}}$) and molecular weight (MW) of benzenoid hydrocarbons.

Dervatives of benzene	BP	E	$\pi -{\text {ele}}$	MW
Benzene	80.1	75.2	8	78.11
Naphthalene	218	141	13.683	128.17
Phenanthrene	338	202.7	19.448	178.23
Anthracene	340	222.6	19.314	178.23
Chrysene	431	271.1	25.192	228.3
Benzo[a]anthracene	425	277.1	25.101	228.3
Triphenylene	429	275.1	25.275	228.3
Tetrcene	440	310.5	25.188	228.3
Benzo[a]pyrene	496	296	28.222	252.3
Benzo[e]pyrene	493	289.9	28.336	252.3
Perylene	497	319.2	28.245	252.3
Anthanthrene	547	323	31.253	276.3
Benzo[ghi]perylene	542	326.1	31.425	276.3
Dibenzi[a,c]anthracene	535	348	30.942	278.3
Dibenzo[a,h]anthracene	535	335	30.881	292.4
Dibenzo[a,j]anthracene	531	336.3	30.88	281.3
Picene	519	336.9	30.943	278.3
Coronene	590	296.7	34.572	300.4
Dienzo[a,h]pyrene	596	375.6	33.928	302.4
Dienzo[a,i]pyrene	594	366	33.954	302.4
Dienzo[a,l]pyrene	595	393.3	34.031	302.4
Pyrene	393	221.3	22.506	202.25

**Table 2:** The values of K-Banhatti indices for benzenoid hydrocarbons.

Dervatives of benzene	$B_1$	$B_2$	$HB_1$	$HB_2$	${}^mB_1$	${}^mB_2$	*HB*
Benzene	48	48	192	192	3	3	6
Naphthalene	92	108	436	660	4.4667	4.1111	8.9333
Phenanthrene	164	218	884	1790	6.5571	5.6667	13.114
Anthracene	164	216	876	1704	6.5048	5.5556	13.01
Chrysene	222	304	1234	2632	8.3619	7.0556	16.724
Benzo[a]anthracene	222	302	1226	2546	8.3095	6.9444	16.619
Triphenylene	222	306	1242	2718	8.4143	7.1667	16.829
Tetrcene	222	300	1218	2460	8.2571	6.8333	16.514
Benzo[a]pyrene	264	374	1520	3410	9.1667	7.4444	18.333
Benzo[e]pyrene	264	376	1528	3496	9.219	7.5556	18.428
Perylene	264	376	1528	3496	9.219	7.5556	18.428
Anthanthrene	306	444	1806	4188	9.9714	7.8333	19.943
Benzo[ghi]perylene	306	446	1814	4274	10.024	7.9444	20.048
Dibenzi[a,c]anthracene	280	390	1584	3474	10.167	8.4444	20.333
Dibenzo[a,h]anthracene	280	388	1576	3388	10.114	8.333	20.229
Dibenzo[a,j]anthracene	280	388	1576	3388	10.114	8.333	20.229
Picene	280	390	1584	3474	10.167	8.4444	20.3333
Coronene	348	516	2100	5052	10.829	8.3333	21.657
Dienzo[a,h]pyrene	322	460	1870	4252	10.971	8.8333	21.943
Dienzo[a,i]pyrene	322	460	1870	4252	10.971	8.8333	21.943
Dienzo[a,l]pyrene	322	462	1878	4338	11.024	8.9444	22.048
Pyrene	206	288	1170	2568	7.3619	6.0556	14.724

**Table 3:** The regression model of statistical parameters for $B_1$.

Regression statistics	BP	E	$\pi$-ele	MW
*R*	0.9901	0.9329	0.9943	0.9862
$R^2$	0.9803	0.8703	0.9887	0.9727
Adjusted $R^2$	0.9793	0.8638	0.9881	0.9713
Standard error	18.5511	28.2573	0.7477	10.0515
F	995.2537	134.2239	1758.2201	712.6998

**Table 4:** The regression model of statistical parameters for $B_2$.

Regression statistics	BP	E	$\pi$-ele	MW
*R*	0.9826	0.9154	0.9876	0.9765
$R^2$	0.9655	0.8380	0.9754	0.9535
Adjusted $R^2$	0.9638	0.8299	0.9741	0.9512
Standard error	24.5458	31.5862	1.1065	13.1130
F	559.9144	103.4296	792.0494	410.5141

**Table 5:** The regression model of statistical parameters for $HB_1$.

Regression statistics	BP	E	$\pi$-ele	MW
*R*	0.9824	0.9149	0.9874	0.9762
$R^2$	0.9651	0.8371	0.9749	0.9530
Adjusted $R^2$	0.9633	0.8289	0.9737	0.9506
Standard error	24.6967	31.6731	1.1160	13.1913
F	552.8501	102.7534	778.3790	405.4172

**Table 6:** The regression model of statistical parameters for $HB_2$.

Regression statistics	BP	E	$\pi$-ele	MW
*R*	0.9658	0.8852	0.9725	0.9569
$R^2$	0.9328	0.7836	0.9457	0.9156
Adjusted $R^2$	0.9294	0.7728	0.9430	0.9114
Standard error	34.2647	36.5008	1.6426	17.6709
F	277.5932	72.4290	348.5135	217.0700

**Table 7:** The regression model of statistical parameters for ${}^mB_1$.

Regression statistics	BP	E	$\pi$-ele	MW
*R*	0.9946	0.9676	0.9983	0.9981
$R^2$	0.9893	0.9362	0.9966	0.9963
Adjusted $R^2$	0.9888	0.9330	0.9964	0.9961
Standard error	13.6686	19.8176	0.4101	3.7155
F	1850.1160	293.5517	5892.7547	5342.4903

**Table 8:** The regression model of statistical parameters for ${}^mB_2$.

Regression statistics	BP	E	$\pi$-ele	MW
*R*	0.9848	0.9767	0.9888	0.9931
$R^2$	0.9698	0.9539	0.9777	0.9862
Adjusted $R^2$	0.9683	0.9516	0.9766	0.9855
Standard error	22.9533	16.8434	1.0536	7.1488
F	643.1725	414.0652	875.6444	1428.5412

**Table 9:** The regression model of statistical parameters for *HB*.

Regression statistics	BP	E	$\pi$-ele	MW
*R*	0.9946	0.9676	0.9983	0.9981
$R^2$	0.9893	0.9362	0.9966	0.9963
Adjusted $R^2$	0.9887	0.9330	0.9964	0.9961
Standard Error	13.6829	19.8161	0.4103	3.7055
F	1846.2014	293.6022	5885.1531	5371.3260

**Table 10:** Correlation coefficients (*R*) between physicochemical properties of benzene derivatives, and K-Banhatti indices.

*PAD*	$B_{1}(G)$	$B_2(G)$	$HB_1(G)$	$HB_2(G)$	${}^mB_1(G)$	${}^mB_2(G)$	*HB*(*G*)
*BP*	0.990101	0.982604	0.982388	0.96581	0.994638	0.984805	0.994627
*E*	0.932908	0.915403	0.914916	0.885221	0.967581	0.976690	0.967587
$\pi -ele$	0.994360	0.987608	0.987395	0.972485	0.998307	0.988771	0.998305
*MW*	0.986257	0.976495	0.976210	0.956889	0.998133	0.993072	0.998143

## Triazine based covalent organic frameworks (CoF’s)

The chemical systems that possess discrete number of molecules refers to supra-molecular chemistry. The spatial arrangement of the molecules is responsible for the strength of the forces between them may be weak or strong. These forces may be due to intermolecular, hydrogen bonding, electrostatic charge, and covalent bonding. The feeble and reversible non-covalent interactions between the molecules are examined by the supramolecular chemistry while traditional chemistry examines covalent bonds. Various functions of supramolecular chemistry comprise molecular recognition, protein folding, interlocked molecular architectures, dynamic covalent chemistry, and other phenomena. Because of its interdisciplinary nature, it attracts physicists, biochemists, biologists, environmental scientists, apart from chemists.

**Figure 3:**
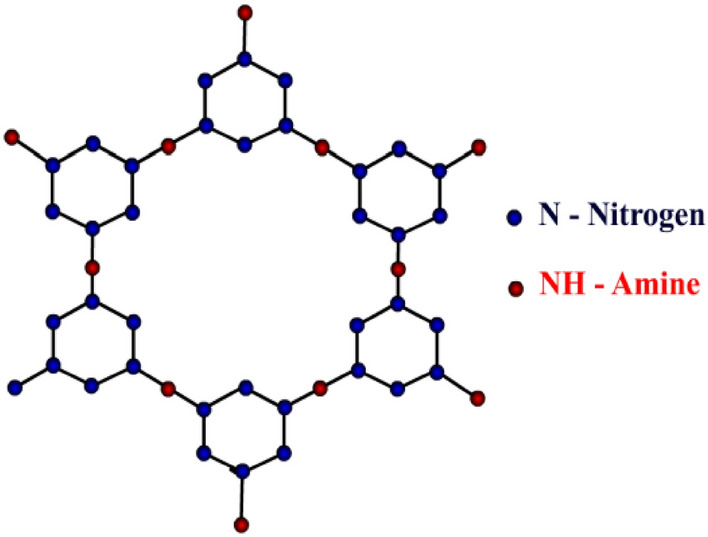
TriCF structure.

This work pinpoints on the supramolecular structure called triazine-based covalent organic frameworks (Fig. 3). To understand better about the chemical and biological properties of a chemical compound, graph theory uses a very useful tool called topological index. These indices help the chemists to derive information about the compound that may be in turn useful in drug design or drug delivery. Chemical graph theory is a combination of chemistry and mathematics in which the compound under the study will be modelled as a graph and the information about its atoms and their bonds are better understood. Chemical graph theory is the result of the strong linkages between both the subjects which have the outcomes as various significant investigations^29–33^.

Biologically significant organic molecules have a new dimension as triazines act as the building blocks used in its design. Triazines and its derivatives have varied applications in antifungal, anticancer, antiviral, cardiotonic, anti-HIV, analgesic, etc., with fine tuned electronic properties. The goal of scientific researchers is to apply their theoretical research in industrial applications so that it is useful for humankind. The objective is to make the products scalable and satisfy excellent properties obtained from the experiments at a reasonable cost and long-term stability.

Covalent organic frameworks (CoF’s) have attracted various researchers across the globe because of its excellent properties such as adsorption, chemo-sensing, energy storage and production. As the CoFs and their applications are found in industries, many research achievements have come to light recently^34–37^. CoF’s may be classified into boron-containing, triazine-based, imide-linked and imine-based due to swift increasing requirements in various fields. We focus on the second category of CoF’s, i.e., triazine-based in this study. In 2008, Thomas et al.^38^, prepared triazine based CoF, by cyclotrimerization of nitrile building units at 400 $^\circ$C in the presence of ZnCl$_2$. There was destruction of ordered structure, despite the harsh conditions during the preparation which included high reaction temperature and purification in acid solution. However, few triazine-based CoF’s show crystallinity, and these building blocks were unable to adapt to harsh temperatures. Later, triazine based CoF’s (CTFs) were synthesized by the condensation reaction of aldehydes and amidines^39^.

The distinguishing physical and chemical features of CoF’s have led to the plethora of applications in the industries. In 2011, Ding et al. reported first set of CoF’s that are useful in the field of catalysis^40^. It was noted that 2-dimensional CoF’s acts as catalyst in different reactions that include nitrophenol reduction, water oxidation, in reducing CO$_2$ to CO etc. CoF’s are used tackle the problem of excessive CO$_2$ emissions as they are the principal reason for greenhouse effect. It is mainly due to the expansion of population and the development of industries. Aqueous alkanolamine is proposed to implement the CO$_2$ emissions. To control the CO$_2$ emissions, new materials are to be developed with high performance in which CoF’s play a significant role. Also, CoF plays an important role in energy storage^41,42^.

Augustine et al.^43,44^ theoretically examined triazine-based covalent-organic frameworks (CoF’s) using vertex and edge partition for degree-based and neighborhood degree-based topological indices. Additionally, the degree-based and neighborhood degree-based entropy measures for the results are given. The graph theoretical approach is used to compare the outcomes with obtained results. In this section, based on the previous work of Tony Augustine et al. various K-Banhatti topological indices are computed for triazine-based covalent-organic frameworks (TriCF).

### Theorem 3.1

Let *G* be the the molecular graph of linear chain TriCF. Then,$B_1(G)$, $B_2(G)$, $HB_1(G)$, $HB_2(G)$, ${}^mB_1(G)$, ${}^mB_2(G)$, and *HB*(*G*) of a graph *G* are

$$\begin{aligned} B_1(G)= 390n+186, \ B_2(G)= 526n+242, \ HB_1(G)= 2142n+990, \ \\ HB_2(G)= 4058n+1798, \ {}^mB_1(G)= \frac{203n+109}{15}, \ {}^mB_2(G)= \frac{97n+59}{9}, \end{aligned}$$

and $$ HB(G)= \frac{406n+2187}{15}. $$

**Figure 4:**
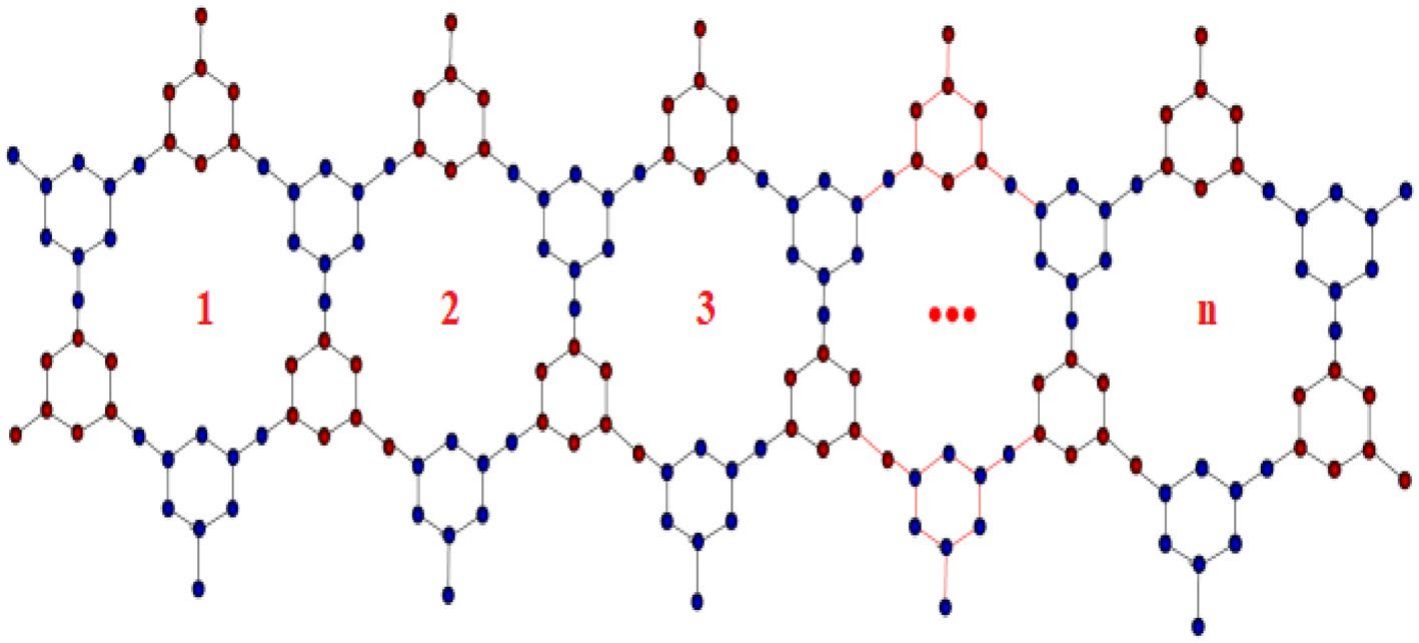
Linear chain of TriCF structure.

### Proof

From the Fig. 4, it is observed that, in general $\vert V(G)\vert =33n+15$ and $\vert E(G)\vert =36n+18$. Also, the edge set of linear chain of TriCF structure is classified into two edge partitions depending on the vertex degrees are given by (see^44^)

$$\begin{aligned}&E_{1,3}=\lbrace uv \in E(G)|d(u)=1, d(v)=3\rbrace ,\\&E_{2,3}=\lbrace uv \in E(G)|d(u)=2, d(v)=3\rbrace , \end{aligned}$$

such that

$$\begin{aligned} E_{1,3}=2n+4, \,\,\ E_{2,3}=34n+14. \end{aligned}$$

We have by the definition of first K-Banhatti index $B_1(G)$ is given by

$$\begin{aligned} B_1(G)&=\sum _{ue}\left[ d_{G}(u)+d_{G}(e)\right] , \quad \quad where,\,\ d_e=d_u+d_v-2\\&= \sum _{ue\in E_{1,3}}\left[ (d_{G}(u)+d_{G}(e))+(d_{G}(v)+d_{G}(e))\right] \\&\quad +\sum _{ue\in E_{2,3}}\left[ (d_{G}(u)+d_{G}(e))+(d_{G}(v)+d_{G}(e))\right] \\&= (2n+4)\left[ (1+2)+(3+2)\right] +(34n+14)\left[ (2+3)+(3+3)\right] \\&=390n+186, \\ \end{aligned}$$

similarly,

$$\begin{aligned}{} & {} B_2(G)= 526n+242, \ HB_1(G)= 2142n+990, \ HB_2(G)= 4058n+1798, \\{} & {} {}^mB_1(G)= \frac{203n+109}{15}, \ {}^mB_2(G)= \frac{97n+59}{9}, \ HB(G)= \frac{406n+2187}{15}. \end{aligned}$$

$$\square $$

### Theorem 3.2

Let *G* be the the molecular graph Parellelogram TriCF. Then $B_1(G)$, $B_2(G)$, $HB_1(G)$, $HB_2(G)$, ${}^mB_1(G)$, ${}^mB_2(G)$, and *HB*(*G*) of a graph *G* are

$$\begin{aligned}&B_1(G)= (198n+192)n+192m-6 ,\ \ B_2(G)= (270m+256)n+256m-14, \\&HB_1(G)= (1098m+1044)n+1044m-54, \ \ HB_2(G)= (2016m+1952)n+1952m-154, \\&{}^mB_1(G)= \frac{(99m+104)n+104m+5}{15}, \ \ {}^mB_2(G)= \frac{(45m+52)n+52m+7}{9}, \ \ \text {and} \\&HB(G)= \frac{(198m+208)n+208m+10}{15}. \end{aligned}$$

**Figure 5:**
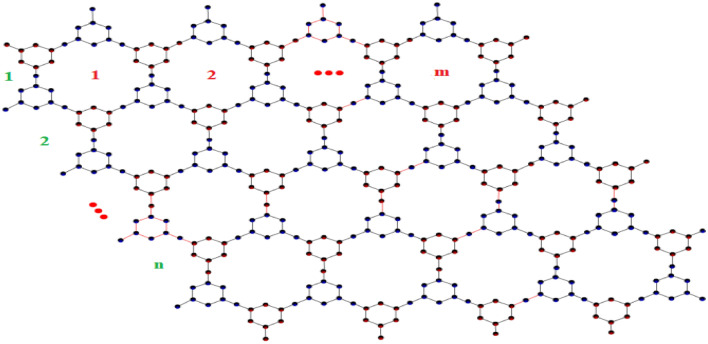
Parallelogram TriCF structure.

### Proof

From the Fig. 5, it is observed that, in general $\vert V(G)\vert =(13m+20)n+3$ and $\vert E(G)\vert =(18m+18)n+18m$. Also, the edge set of parellelogram TriCF structure is classified into two edge partitions depending on the vertex degrees are given by (see^44^)

$$\begin{aligned}&E_{1,3}=\lbrace uv \in E(G)|d(u)=1, d(v)=3\rbrace ,\\&E_{2,3}=\lbrace uv \in E(G)|d(u)=2, d(v)=3\rbrace , \end{aligned}$$

such that

$$\begin{aligned} E_{1,3}=2n+2m+2, \,\ E_{2,3}=(18m+16)n+16m-2. \end{aligned}$$

We have by the definition of first K-Banhatti index $B_1(G)$ is given by

$$\begin{aligned} B_1(G)&=\sum _{ue}\left[ d_{G}(u)+d_{G}(e)\right] , \quad \quad where,\,\ d_e=d_u+d_v-2\\&= \sum _{ue\in E_{1,3}}\left[ (d_{G}(u)+d_{G}(e))+(d_{G}(v)+d_{G}(e))\right] \\ {}&\quad +\sum _{ue\in E_{2,3}}\left[ (d_{G}(u)+d_{G}(e))+(d_{G}(v)+d_{G}(e))\right] \\&= (2n+2m+2)\left[ (1+2)+(3+2)\right] +((18m+16)n+16m-2)\left[ (2+3)+(3+3)\right] \\&=390n+186, \end{aligned}$$

similarly,

$$\begin{aligned}&B_2(G)= (270m+256)n+256m-14, \ \ HB_1(G)= (1098m+1044)n+1044m-54, \\&HB_2(G)= (2016m+1952)n+1952m-154, \ \ {}^mB_1(G)= \frac{(99m+104)n+104m+5}{15}, \\&{}^mB_2(G)= \frac{(45m+52)n+52m+7}{9}, \ \ HB(G)= \frac{(198m+208)n+208m+10}{15}. \end{aligned}$$

$$\square $$

### Theorem 3.3

Let *G* be the the molecular graph Hexagonal TriCF. Then $B_1(G)$,$B_2(G)$, $HB_1(G)$, $HB_2(G)$, ${}^mB_1(G)$, ${}^mB_2(G)$, and *HB*(*G*) of a graph *G* are

$$\begin{aligned}&B_1(G)= 594n^2-18n, \ \ B_2(G)= 810n^2-42n, \ \ HB_1(G)= 3294n^2-162n, \\&HB_2(G)= 6318n^2-462n, \ \ {}^mB_1(G)= \frac{99n^2+5n}{5}, \ \ {}^mB_2(G)= \frac{45n^2+7n}{3}, \\&\text { and }\ \ HB(G)= \frac{198n^2+10n}{5}. \end{aligned}$$

**Figure 6:**
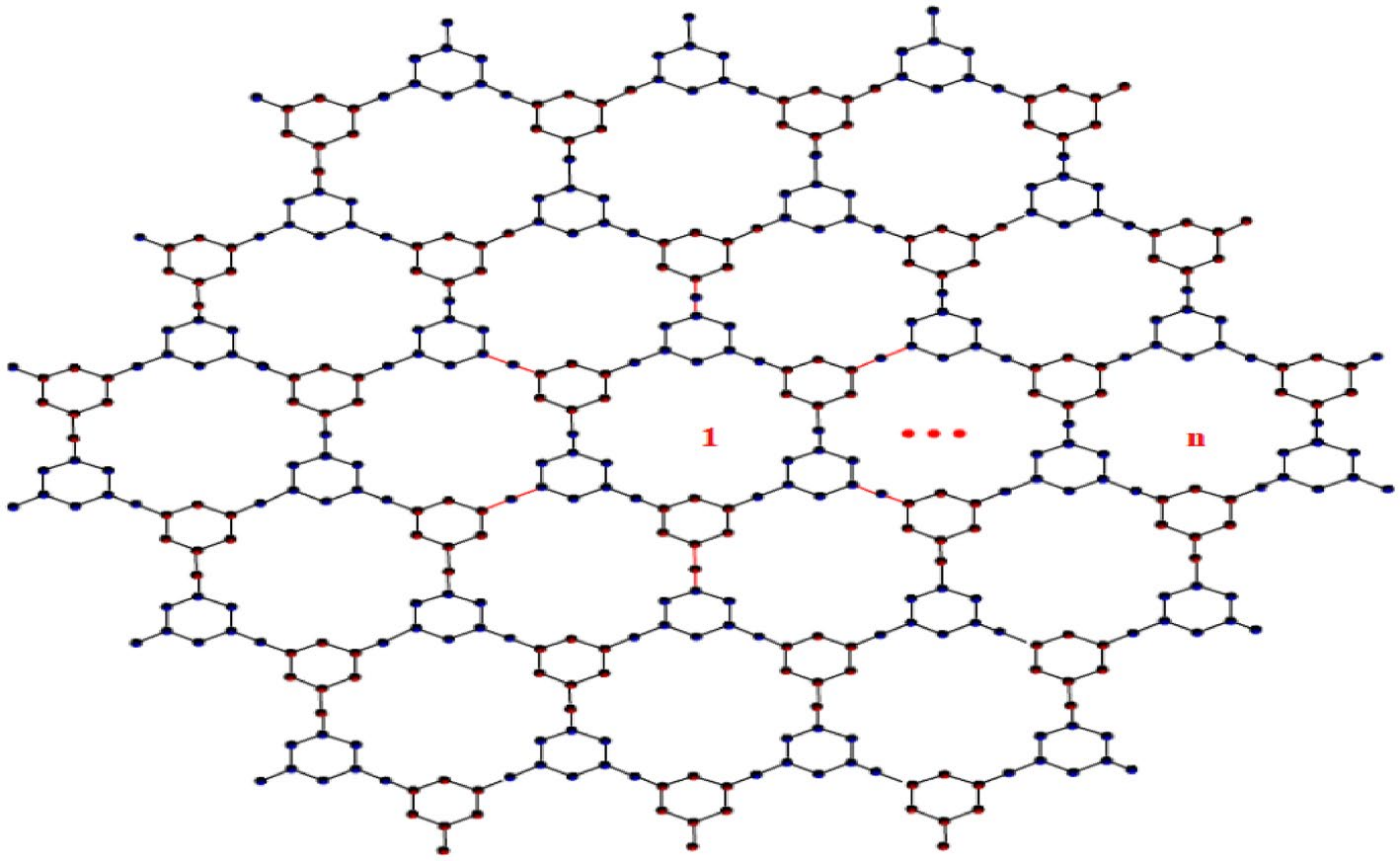
Hexagonal TriCF structure.

### Proof

From the Fig. 6, it is observed that, in general $\vert V(G)\vert =45n^2+3n$ and $\vert E(G)\vert =54n^2$. Also, the edge set of hexagonal TriCF structure is classified into two edge partitions depending on the vertex degrees are given by (see^44^)

$$\begin{aligned}&E_{1,3}=\lbrace uv \in E(G)|d(u)=1, d(v)=3\rbrace ,\\&E_{2,3}=\lbrace uv \in E(G)|d(u)=2, d(v)=3\rbrace , \end{aligned}$$

such that

$$\begin{aligned} E_{1,3}=6n, \,\ E_{2,3}=54n^2-6n. \end{aligned}$$

We have by the definition of first K-Banhatti index $B_1(G)$ is given by

$$\begin{aligned} B_1(G)&=\sum _{ue}\left[ d_{G}(u)+d_{G}(e)\right] , \quad \quad where,\,\ d_e=d_u+d_v-2\\&= \sum _{ue\in E_{1,3}}\left[ (d_{G}(u)+d_{G}(e))+(d_{G}(v)+d_{G}(e))\right] \\ {}&\quad +\sum _{ue\in E_{2,3}}\left[ (d_{G}(u)+d_{G}(e))+(d_{G}(v)+d_{G}(e))\right] \\&= (6n)\left[ (1+2)+(3+2)\right] +(54n^2-6n)\left[ (2+3)+(3+3)\right] \\&=594n^2-18n, \end{aligned}$$

similarly,

$$\begin{aligned}&B_2(G)= 810n^2-42n, \ \ HB_1(G)= 3294n^2-162n, \ \ HB_2(G)= 6318n^2-462n, \\&{}^mB_1(G)= \frac{99n^2+5n}{5}, \ \ {}^mB_2(G)= \frac{45n^2+7n}{3}, \ \ HB(G)= \frac{198n^2+10n}{5}. \end{aligned}$$

$$\square $$

## Numerical and graphical representation and discussion

Figures 4, 5 and 6 showcase the structures of linear chain, Parallelogram and Hexagonal triazine -based covalent oraganic frame works (TriCF) for which the edge and vertex partitions are determined and hence the various forms of K-Banhatti indices are computed.

**Figure 7:**
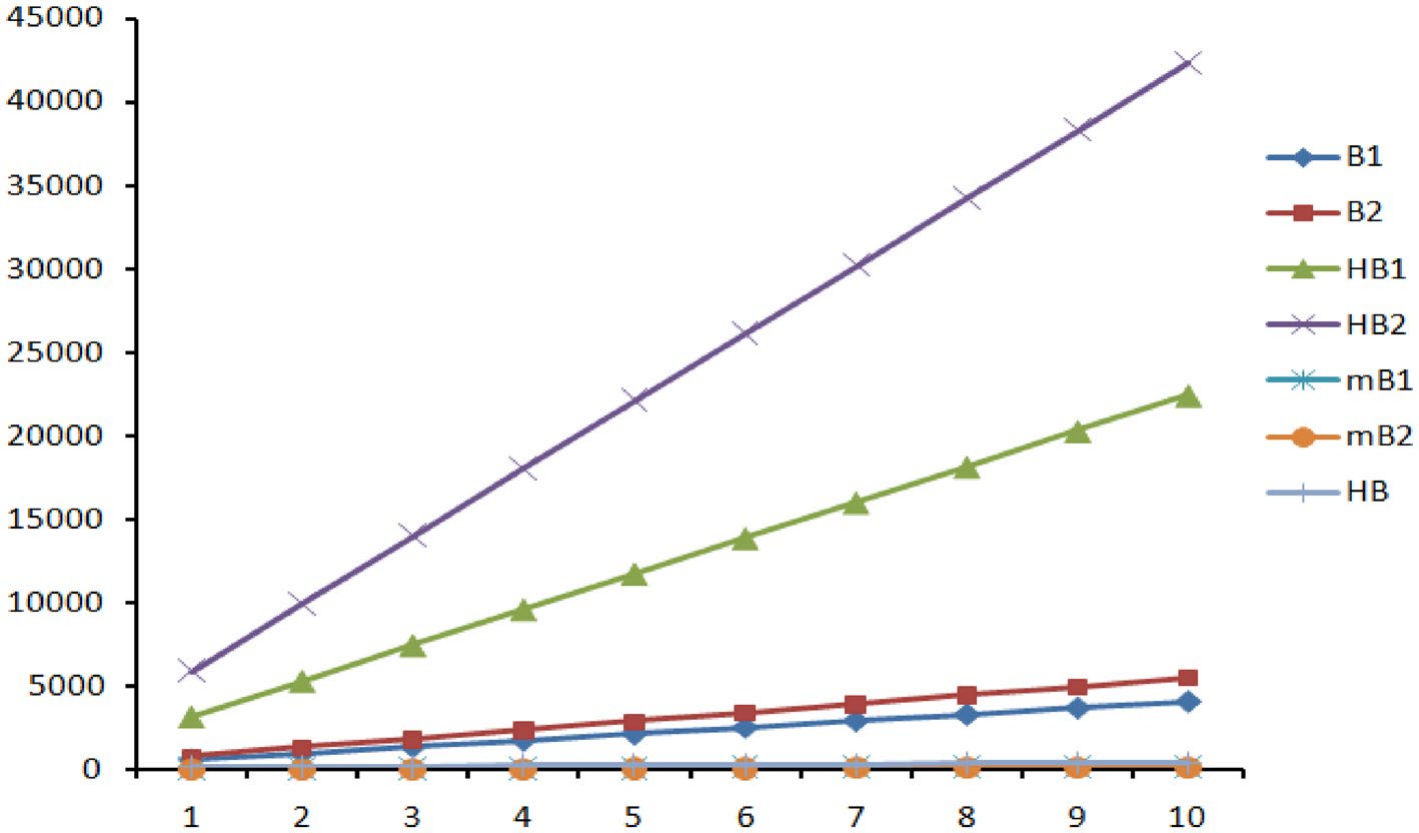
Graphical comparison of K-Banhatti indices for linear chain TriCF, x-axis shows the numeral values of $n\in \{1,2,3,\dots , 10\}$.

**Figure 8:**
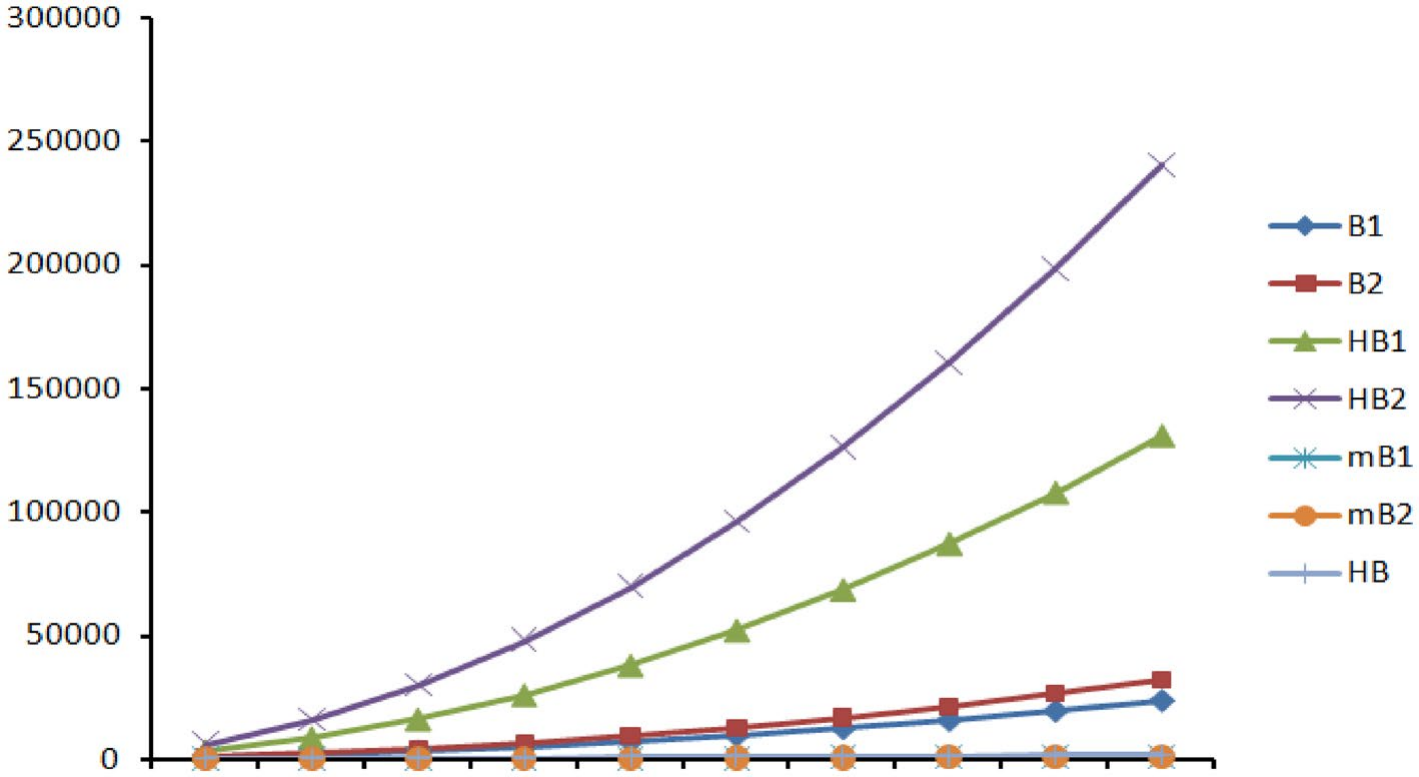
Graphical comparison of K-Banhatti indices for Parellelogram TriCF, x-axis shows the numeral values of $n=m\in \{1,2,3,\dots , 10\}$.

**Figure 9:**
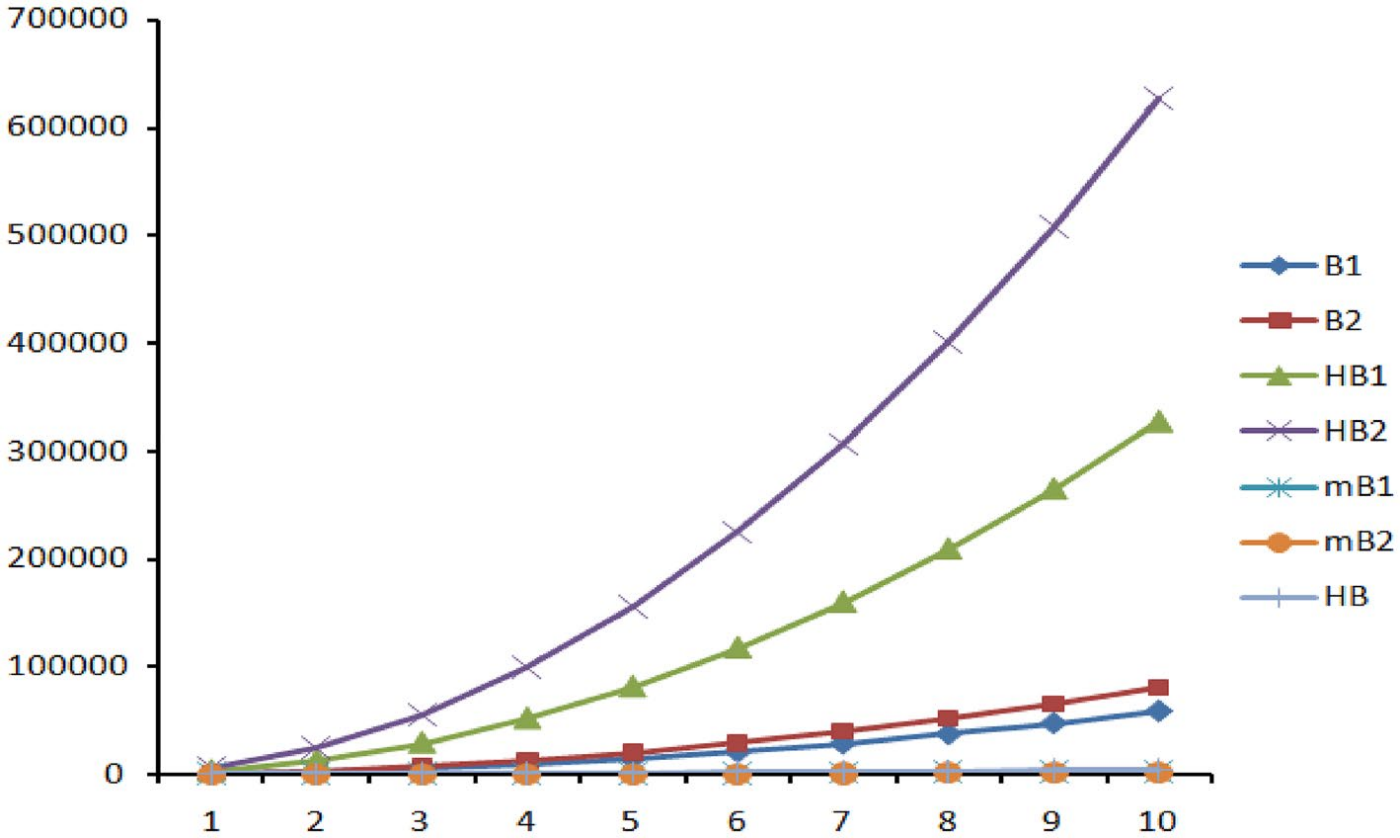
Graphical comparison of K-Banhatti indices for Hexagonal TriCF, x-axis shows the numeral values of $n\in \{1,2,3,\dots , 10\}$.

Figures 7, 8 and 9 represents the graphical comparison of K-Banhatti indices for linear chain TriCF ($n\in \{1,2,3,\dots , 10\}$), Parallelogram TriCF ($n\in \{1,2,3,\dots , 10\}$) and Hexagonal TriCF ($n\in \{1,2,3,\dots , 10\}$) respectively. The figures show that the first K-Banhatti index($B_1$) has more value compared with other K-Banhatti indices while $^mB_1$ and $^mB_2$ showcase the least values and hence it is very close to the x-axis in all the graphs for all triazine-based covalent organic frame works (CoF’s).

Table  11 shows the numerical comparison of K-Banhatti indices for linear chain TriCF structure which linearly increases as n increase. Table  12 shows the variation of the indices under the study for parallelogram TriCF which increases as n, m increase. Finally, Table  13 shows the increase in the indices as n increase.

**Table 11:** The comparison of K-Banhatti indices for linear chain of TriCF structure.

*n*	$B_1$	$B_2$	$HB_1$	$HB_2$	${}^{m}B_{1}$	${}^{m}B_{2}$	*HB*
1	576	768	3132	5856	20.8	17.33	172.87
2	966	1294	5274	9914	34.33	28.11	199.93
3	1356	1820	7416	13,972	47.87	38.89	227
4	1746	2346	9558	18,030	61.4	49.67	254.07
5	2136	2872	11,700	22,088	74.93	60.44	281.13
6	2526	3398	13,842	26,146	88.47	71.22	308.2
7	2916	3924	15,984	30,204	102	82	335.27
8	3306	4450	18,126	34,262	115.5	92.78	362.33
9	3696	4976	20,268	38,320	129.1	103.6	389.4
10	4086	5502	22,410	42,378	142.6	114.3	416.47

**Table 12:** The comparison of K-Banhatti indices for parellelogram TriCF structure.

[*n*, *m*]	$B_1$	$B_2$	$HB_1$	$HB_2$	${}^{m}B_{1}$	${}^{m}B_{2}$	*HB*
[1,1]	576	768	3132	5766	20.8	17.333	41.6
[2,2]	1554	2090	8514	15,718	54.467	43.889	108.93
[3,3]	2928	3952	16,092	29,702	101.33	80.444	202.67
[4,4]	4698	6354	25,866	47,718	161.4	127	322.8
[5,5]	6864	9296	37,836	69,766	234.67	183.56	469.33
[6,6]	9426	12,778	52,002	95,846	321.13	250.11	642.27
[7,7]	12,384	16,800	68,364	125,958	420.8	326.67	841.6
[8,8]	15,738	21,362	86,922	160,102	533.67	413.22	1067.3
[9,9]	19,488	26,464	107,676	198,278	659.73	509.78	1319.5
[10,10]	23,634	32,106	130,626	240,486	799	616.33	1598

**Table 13:** The comparison of K-Banhatti indices for hexagonal TriCF structure.

*n*	$B_1$	$B_2$	$HB_1$	$HB_2$	${}^{m}B_{1}$	${}^{m}B_{2}$	*HB*
1	576	768	3132	5856	20.8	17.333	41.6
2	2340	3156	12,852	24,348	81.2	64.667	162.4
3	5292	7164	29,160	55,476	181.2	142	362.4
4	9432	12,792	52,056	99,240	320.8	249.33	641.6
5	14,760	20,040	81,540	155,640	500	386.67	1000
6	21,276	28,908	117,612	224,676	718.8	554	1437.6
7	28,980	39,396	160,272	306,348	977.2	751.33	1954.4
8	37,872	51,504	209,520	400,656	1275.2	978.67	2550.4
9	47,952	65,232	265,356	507,600	1612.8	1236	3225.6
10	59,220	80,580	327,780	627,180	1990	1523.3	3980

## Conclusion

The molecules are modelled, and their physicochemical and biological properties are predicted using the Quantitative structure-property relationships (QSPR) studies. Topological index is a significant tool used by QSPR studies in encoding the information of a molecule. There are a bunch of topological indices which are of significant importance in the properties of the compounds based on its algorithm defined.

In this article, chemical applicability of $B_1(G)$, $B_2(G)$, $HB_1(G)$, $HB_2(G)$, ${}^mB_1(G)$, ${}^mB_2(G)$, and *HB*(*G*) for benzenoid hydrocarbons of a graph G are examined and it is observed that the considered indices (molecular descriptors) showed good predictive potential. Benzenoid hydrocarbons have numerous applications because of its unique physical and chemical properties. Some of them include paint thinners, laminates, cement, in medicine for curing bacterial infections, mosquito repellents, cosmetics, toothpaste, detergents, and a dyeing agent.

Also the above said indices are computed for triazine-based covalent organic frameworks (CoF’s). Triazine has wide applications in industries, where one of the famous forms being melamine. It is used in kitchen appliances and carpentry. Another form of triazine is cyanuric chloride that are used in reactive dyes and herbicides. It has several applications in oil, petroleum and gas processing industries. They are used to remove harmful hydrogen sulphide gas and other species from fluid streams in infrastructure. As the chemical compound, triazine has many applications especially in industries, the work can be extended for other indices using graph operators and see the variation. Also, it has applications in medical field, attracting the pharmacists and chemists in the usage of drug design and delivery.

## Data Availability

The datasets used and analysed during the current study available from the corresponding author on reasonable request.
